# Proteomic interrogation of the meninges reveals the molecular identities of structural components and regional distinctions along the CNS axis

**DOI:** 10.1186/s12987-023-00473-w

**Published:** 2023-10-19

**Authors:** Elise Santorella, Jeremy L. Balsbaugh, Shujun Ge, Parisa Saboori, David Baker, Joel S. Pachter

**Affiliations:** 1 Department of Immunology, UConn Health, 263 Farmington Ave, Farmington, CT 06030 USA; 2 Proteomics and Metabolomics Facility, Center for Open Research Resources & Equipment, University of Connecticut, Storrs, CT 06269 USA; 3 Department of Mechanical Engineering, Manhattan College, Bronx, NY 10071 USA; 4 Blizard Institute, Queen Mary University of London, London, England

## Abstract

The meninges surround the brain and spinal cord, affording physical protection while also serving as a niche of neuroimmune activity. Though possessing stromal qualities, its complex cellular and extracellular makeup has yet to be elaborated, and it remains unclear whether the meninges vary along the neuroaxis. Hence, studies were carried-out to elucidate the protein composition and structural organization of brain and spinal cord meninges in normal, adult Biozzi ABH mice. First, shotgun, bottom-up proteomics was carried-out. Prominent proteins at both brain and spinal levels included Type II collagen and Type II keratins, representing extracellular matrix (ECM) and cytoskeletal categories, respectively. While the vast majority of total proteins detected was shared between both meningeal locales, more were uniquely detected in brain than in spine. This pattern was also seen when total proteins were subdivided by cellular compartment, except in the case of the ECM category where brain and spinal meninges each had near equal number of unique proteins, and Type V and type III collagen registered exclusively in the spine. Quantitative analysis revealed differential expression of several collagens and cytoskeletal proteins between brain and spinal meninges. High-resolution immunofluorescence and immunogold-scanning electronmicroscopy on sections from whole brain and spinal cord – still encased within bone –identified major proteins detected by proteomics, and highlighted their association with cellular and extracellular elements of variously shaped arachnoid trabeculae. Western blotting aligned with the proteomic and immunohistological analyses, reinforcing differential appearance of proteins in brain vs spinal meninges. Results could reflect regional distinctions in meninges that govern protective and/or neuroimmune functions.

**Supplementary Information:**

The online version contains supplementary material available at 10.1186/s12987-023-00473-w.

## Introduction

The meninges line the skull and vertebral canal, tightly enveloping the brain and spinal cord, respectively, in apparent custodial manner. Yet, despite having been described as early as the third century B.C.E. [30], the meninges still remain largely enigmatic in both function and composition. What is well known is that they are comprised of three, overlapping membranes that are aptly named in light of their distinct gross features and histological presentation (See reviews [Patel and Kirmi, [22, 23, 29, 84] for general descriptions and diagrammatic representations). The dura mater (Latin, *durable mother*) lies directly apposed to the bone (skull and vertebrae), is the thickest and most fibrous of the three, and, more generally, is comprised of an outer periosteal layer and inner meningeal layer (the human dura has an innermost dural layer called the dural border cell layer [55]). Next, is the arachnoid (Latin, *spider-like*), which is formed of two distinct cell layers, and associated subarachnoid space (SAS), the latter traversed by a web of arachnoid projections called trabeculae that extend from the inner layer of arachnoid cells. Within the SAS lie large blood vessels that send microvessel tributaries into the parenchyma, and cerebral spinal fluid (CSF) that percolates through SAS channels fashioned by the walls of the anastomosing trabeculae. The pia mater (Latin, *protective mother*) is the innermost membrane, closely affixed to the parenchyma, and appears continuous with the trabeculae and arachnoid cells.

By virtue of keeping the brain and spinal cord buoyed in CSF, the meninges have been conceived as principally serving a protective role (Patel and Kirmi, [67, 84, 114, 120]. However, other responsibilities beyond the mechanical realm have begun to emerge. Included among these additional roles are secretion of several trophic factors, generation of neurons from progenitors, generation and formation of the glia limiters, and regulation of cell migration and vascularization [29]. Burgeoning data also point to the meninges as a cradle of immune activity in a wide host of neurologic diseases with inflammatory involvement, including – but not exclusive to – multiple sclerosis (MS, Alzheimer’s disease, cerebral amyloid angiopathy, migraines, trauma and stroke (Russi and Brown, [96, 95], de Lima et al., 2020). In this capacity, the meninges can serve as a launching pad from which inflammatory impulses and/or the signals that elicit them propagate down into the parenchyma [17, 73, 110].

Likewise, there is increasing awareness of the complexity of the make-up and organization of meningeal components. The arachnoid trabeculae are the most elaborate, and remain perhaps the most cryptic. As viewed by scanning electron microscopy (SEM), trabeculae manifest several morphologies, having variously been described as “tree-like,” “veil-like,” and “rod-like” (Saboori, [111]). And, in what may reflect still other forms of trabeculae, continuous structures referred to as “membranous septa” [4, 22, 23, 77] and sheet-like “cisterns” [86] have been seen coursing through the SAS in transmission electron and light microscopy images. Even the term “meningothelial cells,” to refer to those cells lining the arachnoid, pia mater and arachnoid trabeculae [36, 126], imparts a compositional vagueness to these elements. Such various characterizations contribute to the ambiguity of the meninges, and underscore the need for more clarification of structure–function relationships. In this regard, the composition and labyrinthine nature of the trabeculae may hold particular significance for organizing and guiding immune activities and/or developmental processes [66].

But fuller characterization of the adult meninges has been frustrated by technical challenges that have hampered keeping the intricate cytoarchitecture of this tissue intact during routine histological preparation, and impeded assigning molecular and cellular identities to meningeal components. For example, maintaining integrity of the meninges for histological analysis is highly problematic due to the delicate attachment of this tissue to bone. Craniectomy and laminectomy to access the brain and spinal cord, respectively, can significantly disrupt the intimate arrangement of the meningeal membranes and the three-dimensional order of their components. Particularly susceptible to damage is the delicate reticulum of trabecular structures [78]. On the other hand, efforts to section adult CNS tissue in situ, typically require decalcification of the heavily mineralized cranium and vertebrae by caustic agents that can also cause tissue destruction and attenuate immunoreactivity [5, 63]. These structural obstacles aside, targets for immunodetection of the meninges are sparse, as the protein composition of the adult, normal meninges has yet to be elaborated, the focus, instead, being on meningiomas [1, 82, 112]. Likewise, while differences in the meninges between brain and spinal cord have been implied [74], they have never been detailed – a gap in the literature perhaps due to yet other investigational hurdles. An elaborate molecular characterization of the meninges at different CNS sites has, thus, been elusive.

Accordingly, a bifurcated approach was taken to elucidate the protein composition and structural organization of the meninges at both the brain and spinal cord level in normal, adult Biozzi ABH mice, with a focus on the reticular network of arachnoid trabeculae. First, shotgun proteomics was performed separately on brain or spinal meninges. Following bioinformatic interrogation to identify major structural proteins, high-resolution immunofluorescence imaging and immunogold-SEM were performed on sections of intact, whole brain and spinal cord still encased within the skull and vertebral column, respectively. Proteomic results indicated that while the protein repertoires of brain vs spinal cord meninges were largely similar, several proteins were differentially expressed to a significant extent at the respective locales. Immunostaining further identified specific collagens associated with varied forms of arachnoid trabeculae, as well as clarified cell types that attend these structures. These findings are interpreted in terms of factors that might dictate immune regulation along the CNS axis, causing brain or spinal cord to be preferentially affected during neuroinflammatory disease.

## Materials and methods

### Mice

Male and female Biozzi ABH H-2 (H-2^dq1^) mice were originally obtained from Harlan UK Ltd, Bicester, UK, and bred at Queen Mary University of London, under pathogen-free conditions. A breeder pair was then used to establish another colony at UConn Health, Farmington, CT. Mice, age 8—10 weeks, were used throughout. All mice were maintained under specific pathogen-free conditions and all animal protocols were following Animal Care and Use Guidelines of UConn Health (Animal Welfare Assurance # A3471-01).

### Contrast staining of the meninges with Evan’s Blue

To eliminate as much as possible the contribution of serum proteins to the meningeal proteomes, mice were exsanguinated by cardiac perfusion. Because removal of the blood renders the meninges transparent, immediately following exsanguination mice were perfused with Evan’s blue dye to highlight the meningeal vasculature and clearly distinguish the meninges from the underlying parenchyma (Additional file 1: Fig. S1). Specifically, mice were anesthetized with 300 μL ketamine (100 mg/ml, Zoetis, USA)/xylazine (20 mg/ml, Akorn Animal Health, USA) and initially perfused with 10 mL heparin (Sigma-Aldrich, USA)/phosphate buffered saline (PBS) pH 7.4, (140u heparin [Sigma-Aldrich]/mL PBS). Mice were subsequently perfused with 10 mL of a 0.5% Evans Blue (Sigma-Aldrich) in 1X PBS for contrast, followed by another 5–10 mL of heparin/PBS to wash out unbound dye.

### Dissection and protein extraction

The protocol for dissecting the brain meninges largely followed the extremely detailed procedure described by Derecki and Kipnis, 2014 (10.1038/protex.2014.030), with the exception that, after making the incisions, the large bones were not removed. Instead, each cut bone fragment was gently lifted while the attendant dura was grasped with forceps. This was repeated until the brain was freed of all skull attachments. Regarding the spinal cord meninges, the entire spinal column was removed and cleaned of flesh from all aspects of the vertebrae. The column was then cut into 3–4 sections, slicing each section on a slight diagonal for ease of dissection. Within each section, the vertebrae were slowly pulled apart exposing the underlying meninges. Meninges were grasped with #5 forceps and gently peeled off the spinal cord. The spinal cord was kept hydrated with PBS to prevent the meninges from sticking to the underlying parenchyma. As with the brain, the Evan’s blue staining of the meninges kept this tissue clearly discernable from the parenchyma underneath.

An extraction buffer consisting of a 1:9 solution of protease inhibitor cocktail (Sigma-Aldrich) in Pierce™ RIPA buffer (Thermo Fisher Scientific, USA) was prepared, and 150–200 μl aliquoted into separate 1 ml Dounce glass homogenizers (custom-ordered from PerkinElmer, USA) kept on ice. RIPA buffer was selected for meningeal extraction as it contains both NP40 (nonionic) and sodium deoxycholate (ionic) detergents and, thus, is sufficiently chaotropic to allow effective solubilization of cytoplasmic, nuclear and membrane proteins, generating a whole cell lysate most representative of the respective brain and spinal cord meningeal compartments. Following craniectomy and laminectomy, brain and spinal cord meninges, respectively, were removed to their individual homogenizers and manually dispersed, first using the looser fitting A pestle (clearance ~ 0.0025–0.0055 in.), then the tighter fitting B pestle (clearance ~ 0.0005—0.0025 in.). Care was taken to scrape the skull and vertebrae for all traces of dura, while avoiding capturing any small bone shards. Meninges from two mice were used per sample. Following homogenization, the separate samples of brain and spinal cord meninges were placed in 1.5 mL centrifuge tubes and spun at 14,000 × g for 20 min. Supernatants were then transferred to 0.22 μm centrifuge tube filters (Corning, USA), and spun for an additional 5 min at 14,000 × g. Resulting filtrates were removed, aliquoted, and stored at − 80 °C until assayed. Before proteomics, protein concentration of samples was determined by Pierce™ Rapid Gold BCA Protein Assay Kit (Thermo Fisher Scientific).

### Proteomics

#### Sample preparation for liquid chromatography and tandem mass spectrometry (LC/MS–MS)

Samples of filtered meningeal extract were prepared using a slightly modified Filter-Aided Sample Preparation (FASP) method in a Microcon YM-10 10kD molecular weight cutoff (MWCO) filter (Thermo Fisher Scientific) [124]. First, samples were diluted in UA buffer (8 M Urea, 0.1 M Tris–HCl, pH 8.5) and reduced for 1.5 h at 37 °C using 25 mM dithiothreitol. Fully reduced proteins were concentrated onto the filter and the buffer was spun through the 10 kD MWCO filter at 14,000 × g for 40 min. The proteins and filter assembly were washed a second time with 200 µL UA buffer and spun at 14,000 × g for 40 min. Next, Cys residues were alkylated using 50 mM iodoacetamide in UA buffer for 15 min in the dark at 37 °C, after which the filters were spun at 14,000 × g for 30 min and the flow-through buffer was removed. Two more buffer exchange steps were instituted against 100 µL UB buffer (8 M Urea, 0.1 M Tris–HCl, pH 8.0), each time with identical centrifugation conditions. A simultaneous protein resuspension and filter wash step using 50 µL UB buffer was used to aid removal of the proteins from the filter for placement into clean 1.5 mL Eppendorf tubes. The filter was washed twice to resuspend any remaining proteins with 50 µL aliquots of 0.1 M ammonium bicarbonate and pooled with the first protein aliquot*.* Endoproteinase LysC (Pierce, USA) was added at a 1:50 enzyme: protein ratio and samples left to digest at 37 °C for 16 h. Samples were then diluted to < 1 M urea with 0.1 M ammonium bicarbonate, and sequencing grade modified trypsin (Promega, USA) added at a 1:50 enzyme: protein ratio and left to digest for an additional 8 h at 37 °C. Proteolysis was quenched using formic acid and the resulting peptides desalted using Pierce™ C18 Peptide Desalting Spin Columns (Thermo Fisher Scientific) per manufacturer’s instructions.

#### Untargeted shotgun proteomic analysis by LC/MS–MS

Desalted peptides were injected onto a Waters nanoEase m/z Peptide UPLC BEH C18 column (1.7 µm, 130 Å, 75 µm × 250 cm) and separated using a 300 nL/min nanoflow 180 min reversed phase gradient on a Dionex Ultimate 3000 RSLC UPLC instrument (Thermo Fisher Scientific). The Ultimate 3000 UPLC was coupled directly to a Q Exactive HF mass spectrometer (Thermo Fisher Scientific) and eluted peptides were subject to nanoflow electrospray ionization and direct entry into the mass spectrometer. The Q Exactive HF was operated in positive mode using a Top 15 data-dependent MS/MS acquisition method.

#### Data processing

All raw files were searched against the Uniprot *Mus musculus* reference proteome database (Reference proteome UP000000589, accessed October 18, 2020) using the Andromeda search engine embedded in MaxQuant (v1.6.1.0) [25]. The following parameters were used for peptide/protein identification: 1% False Discovery Rate (FDR) at the protein and peptide levels, variable modifications include oxidized Met, acetyl protein N-terminus, N-terminal peptide Gln to pyro Glu, and deamidation of Asn and Gln. Fixed carbamidomethylation on Cys residues, a minimum value of 5 amino acid per peptide, and trypsin digestion specificity with 2 missed cleavages were also employed. All other parameters were kept at default values.

The mass spectrometry data have been deposited to the Proteome Xchange Consortium via the PRIDE partner repository with the dataset identifier PXD039294. Reviewers can access the private dataset using usernamereviewer_pxd039294@ebi.sc.ukand temporary password 5QKqHT2A.

#### Data analysis

Peptide and protein quantification were performed by the MaxQuant LFQ algorithm. All search results were uploaded into Scaffold v4.10 (Proteome Software, Inc., USA) for visualization and further analysis. High confidence in protein identification was afforded through multiple post-informatics search filters. One, an initial 1% FDR filter using a target-decoy search approach at the protein and peptide level was employed to limit the number of false positive identifications. Two, a strict two unique peptide sequences per protein experiment-wide filter in Scaffold was required for identification. Three, biological replicate thresholding was imposed, requiring positive identification in each replicate experiment. Power analysis calculations indicated increasing from N = 3 to N = 5 replicates gave reasonable power while greatly decreasing the type I error rate. Replicates were further increased to N = 6.

The Average Precursor Intensity (API) quantitative unit as a measure of total protein abundance was used directly to gauge protein-level differences both within and across proteomics experiments. Data files containing quantitative values were exported to Microsoft Excel, R, or Python for different enrichment analyses. A threshold for frequency of protein detection was implemented to underscore reproducibility. Specifically, a protein was considered to be a legitimate component of the meningeal proteome if it was detected in 4 or more brain or spinal cord samples.

Gene Ontology (GO) Cellular Component designations and enrichment data were exported using the PSEA-Quant algorithm of Scaffold (Lavellee-Adam et al. [62]) and used to generate Venn Diagrams; proteins were organized and grouped depending on their membership to eleven Cellular Component designations highlighted in Scaffold (*Cytoplasm, Cytoskeleton, Endoplasmic Reticulum, Endosome, Extracellular Region, Golgi Apparatus, Mitochondrion, Nucleus, Plasma Membrane, Extracellular Matrix*, and *Ribosome*).

#### Enrichment analysis

Enrichment analysis was performed to determine statistically significant differences in the frequency of GO terms overrepresented in the meningeal proteome. GO term repositories are informed by peer-reviewed experimental data, scientific literature, and functional annotations [10].

Protein lists were filtered as described in “Data Analysis” and organized into three subsets: “Brain Proteome” (proteins shared between brain and spinal cord meninges plus proteins expressed above-threshold only in brain meninges), “Spinal Cord Proteome” (proteins shared between brain and spinal cord meninges plus proteins expressed above-threshold only in spinal cord meninges), and “Shared Only” (only proteins shared between brain and spinal cord meninges). Each data subset consisted of a list of proteins (present in at least 4/6 samples) and their respective Uniprot Accession Numbers (UniprotAN). UniprotANs for each data subset were imported into GOnet (https://tools.dice-database.org/GOnet/) to obtain enrichment and annotation data for one of three Gene Ontology categories: Cellular Component, Biological Process, and Molecular Function. GOnet enrichment exports include lists of GO terms and their corresponding p-values.

Gene Ontology annotation and enrichment data for Cellular Component, Biological Process, and Molecular Function was obtained for each sub-divided data set through GOnet (https://tools.dice-database.org/GOnet/). Lists of significantly enriched GO terms and their corresponding p-values (p ≤ 0.05) were then analyzed using ReViGO’s default parameters (revigo.irb.hr). ReViGO Tree Map data was exported, manually cross-checked for correct parent-term assignment using QuickGO (https://www.ebi.ac.uk/QuickGO/annotations), and imported into CirGO to generate two-tier enrichment pie charts.

#### Volcano Plot

The volcano plot was generated in R using the following packages: colorspace, dplyr, e1071, ggplot2, ggrepel, gplots, RColorBrewer, tidyverse. To be included in the volcano plot, a protein had to adhere to the 4 of 6 replicate threshold in at least one meningeal compartment (i.e., brain and/or spinal cord) in the proteomic analysis. Fold change and p-value for each protein were calculated in Microsoft Excel according to the data analysis pipeline described by Aguilan et al. [3].

### Western blotting

Aliquots of meningeal lysates from proteomic samples (10 μg of protein) were diluted with Laemmli sample buffer (BioRad, USA), adjusted to 2% (v/v) β-mercaptoethanol (Sigma Aldrich), and heated at 95 °C for 10–15 min. Following clarification at 14,000 × g for 1 min, samples were then separated by SDS-PAGE on 4–15% Mini-PROTEAN^®^ TGX™ Precast Protein Gels (BioRad). Electrophoresis was initiated at 50–65 V for the first 5 min, then voltage was increased to 100–150 V for the remaining 60–90 min. After electrophoresis was complete, gels were immediately transblotted onto nitrocellulose using the Trans-Blot Turbo system (BioRad). Successful protein transfer was confirmed by incubation of blots with Ponceau S solution (Sigma Aldrich, USA) for 1 min, followed by brief rinsing in distilled H_2_O. Blots were then washed 3 × 5 min in a Tris-buffered saline solution (TBST) with 0.1% Tween^®^ 20 detergent (Sigma Aldrich, USA). TBST and all derivative solutions were filtered through a 40 μm Falcon cell strainer before use (Thermo Fisher, USA). Following washes, blots were incubated in a 5% (w/v) solution of non-fat dry milk (BioRad, USA) in TBST for 1 h. Blots were then washed 3 × 5 min in TBST, and incubated in a 3% (w/v) bovine serum albumin solution containing primary antibody (Additional file 1: Table S1) overnight on a shaker at 4 °C. The following day, blots were removed from primary incubation and washed 3–5 times with TBST. Blots were then incubated with a solution of TidyBlot Western Blot Detection Reagent (BioRad, USA) for 1 h at room temperature, rinsed 3 × 5 min with TBST, and developed with SuperSignal West Pico Chemiluminescent Substrate (Thermo Fisher, USA) for 5 min at room temperature. Development of blots was quenched by a brief rinse in distilled H_2_O, then blots were imaged using a Molecular Imager Gel Doc XR + System with Image Lab Software (BioRad, USA). Subsequent quantitation of blots was performed using Image J (NIH, USA). Relative quantitation was calculated as a signal intensity ratio of each protein band relative to the loading control. For purposes of re-probing, blots were stripped by incubation in Restore™ Western Blot Stripping Buffer (Thermo Fisher, USA) at room temperature for 15 min. Blots were then washed 3 × 5 min with TBST and blocked in 3% BSA solution for an hour at room temperature. Following this blocking step, all additional steps were the same as for initial protein detection.

### Histology

#### Tissue preparation

Mice were anesthetized with 300 µL ketamine/xylazine and perfused with 10 mL heparin-Phosphate Buffered Saline, pH 7.4 (PBS), followed by 10 mL of 4% paraformaldehyde (Electron Microscopy Sciences, USA) in PBS. After perfusion, the entire skull and spinal column were removed and post-fixed in 4% paraformaldehyde at 4 °C for 24 – 48 h (spinal columns requiring more time for penetration of fixative). Samples were then transferred to a solution of 30% sucrose (J. T. Baker, USA) in PBS at 4 °C for cryopreservation. After equilibration, samples were embedded in Cryomatrix (Thermo Fisher Scientific) and stored at − 80 °C until sectioning.

#### Cryosectioning

Sections were cut on a Leica CM 1850 cryostat. Frozen longitudinal sections of whole mouse spinal cord, with vertebrae intact, and frontal and sagittal sections of whole brain, encased within the cranium, were sectioned between 5 and 30 μm thickness (depending on the downstream application) directly onto to an adhesive tape (Cryofilm type II, Section-Lab, Japan), as previously detailed [110]. For fluorescence microscopy and imaging mass cytometry, the tape was then adhered—tissue-side facing up—to a charged glass slide with 1% (w/v) chitosan (Sigma-Aldrich). Slides were next placed on a slide warmer at 37 °C for 30–60 min. Tape edges were further secured to the slides by outlining the tape with rubber cement glue (Pliobond 25, Pliobond, PC-225-LV, Ellsworth Adhesives, USA) and allowed to solidify for 24 h.

#### immunofluorescence/confocal microscopy

Permeabilization, blocking and staining of Sects. (20–30 μm) were performed essentially as described previously [85], Shrestha et al. [111]). Primary and secondary antibodies used (including sources and working dilutions) are listed in Additional file 1: Tables S1 and S2, respectively. After antibody staining, nuclei were labeled with 0.1% (v/v) DAPI (Sigma-Aldrich) in PBS for 5 min, followed by washing of sections with 0.05% (v/v) Tween^®^ 20 (Sigma-Aldrich) in PBS. Sections were then allowed to air-dry for 5–10 min, and mounted in Mowiol (Sigma-Aldrich). Confocal z-stacks (multi-track scan) were acquired using a Zeiss LSM 780, with the Zen Blue Edition software system (Carl Zeiss Microscopy, LLC, USA), and 10x/0.45 W C-Apochromat, 20x/0.8 Plan-Apochromat, and 40x/1.2 W C-Apochromat. (Carl Zeiss Microscopy, LLC) lenses. Thereafter, z-stacks were imported into Bitplane Imaris^®^ suite version 9.2.1. software (Bitplane Inc., USA).

#### Imaging mass cytometry (IMC)

Maximal thickness of sections was 10 μm. Permeabilization and blocking steps were as for immunofluorescence. Metal-conjugated primary antibodies (Standard BioTools Inc., USA) were used throughout. To ensure that the signal of each metal-conjugate did not interfere with another, Maxpar^®^ Panel Designer (Maxpar Panel Designer v2.0, DVS Sciences and Standard BioTools Inc.) was employed. All primary antibodies were combined in a single cocktail in 0.5% Bovine Serum Albumin (BSA) (Sigma-Aldrich) in PBS. Slides were incubated with primary antibodies overnight at 4 °C. Following antibody incubation, slides were washed with PBS and stained with Cell-ID™ Intercalator-Ir (Standard BioTools Inc.) and OsO_4_ (Sigma-Aldrich) in PBS for 2—3 h to label DNA and lipids, respectively, then washed with distilled, deionized water twice for 5 min before air-drying.

IMC data was obtained using the Hyperion™ imaging system in tandem with a Helios™ Mass Cytometer (Standard BioTools Inc.). Briefly, samples stained with metal-conjugated antibodies were scanned and vaporized by laser-ablation in 1 μm^2^ increments. The resulting vaporized samples were captured by the mass cytometer and analyzed using time-of-flight mass spectrometry (CyTOF) (Standard BioTools Inc.). Individual signals for each isotope detected in a sample were resolved and compiled with time-of-flight mass spectrometry data into a high-dimensional.mcd image file of the target area.

#### Immunoelectron microscopy

Permeabilization, blocking and staining of Sects. (10–20 μm) were performed as for immunofluorescence/confocal microscopy, with the exception that the diluent used was 0.1 M Tris-buffered saline, pH 7.4. The change in buffer was to ensure compatibility with FluoroNanoGold™ secondary antibodies Alexa Fluor^®^ 488—FluoroNanogold™ Fab' rabbit anti-goat IgG (H + L) and Alexa Fluor^®^ 594**—**FluoroNanogold™ Fab' goat anti-mouse IgG (H + L) (Nanoprobes Inc., USA) and later gold enhancement. Gold enhancement was performed using the GoldEnhance™ EM Plus enhancement kit (NanoProbes). Immunogold labeled/gold enhanced spinal cord sections were fixed in 1% glutaraldehyde in 0.1 M cacodylate buffer for 30 min, then rinsed three times in 0.1 M sodium cacodylate buffer, pH 7.4. Samples were post-fixed with 1% OsO_4_ /0.8% C_6_N_6_FeK_3_ in 0.1 M cacodylate buffer for 1 h, then rinsed five times in distilled water. Next, samples were dehydrated in 50%, 75%, 95% ethanol and three changes of 100% ethanol for at least 10 min per solution. Samples were further dehydrated in 1:2, then 2:1 hexamethyldisilazane (HMDS, electron microscopy sciences): ethanol for 20 min per solution, then in two changes of 100% HDMS, remaining in 100% HMDS overnight. The sections were trimmed and attached to a 1 cm diameter SEM stub with double sided carbon tape (electron microscopy sciences), then sputter coated with gold target in a Denton Vacuum Desk V for 60 s. Samples were imaged on a Jeol JSM5900LV, in secondary electron or backscatter mode.

## Results

### Proteomes of brain vs spinal meninges

Total proteins detected in brain and spinal meninges are listed in Additional file 2: Table S3. Bottom-up proteomic interrogation (Chait, [19]) indicated that Type II collagen, alpha-1 (Col2A1) and intermediate filament (IF)-forming Type II keratins (keratin 76 or keratin 8) were among the twenty-five most abundant proteins recognized at both locales. Specifically, Type II collagen was prominent at both locales, while keratin 76 was prevalent in brain, and keratin 8 in spinal cord (Table 1). The substantial meningeal presence of Type II collagen coincides with the depiction of the arachnoid trabeculae as “spongy connective tissue made of collagen fibers and fibroblasts” [29]. Expression of Types II keratins, on the other hand, is in line with several descriptions in the literature referring to the arachnoid as possessing epithelial qualities [18, 94].

**Table 1:** The 25 most abundant proteins in brain and spinal cord meninges

Brain	Spinal Cord
Predicted gene 17087	Predicted gene 17087
**Type II Collagen, alpha 1**	Inactive phospholipase (Fragment)
Nucleoredoxin	Myosin light chain 4 (Fragment)
Myosin light chain 6B	Parvalbumin alpha
Histone H1.3	Beta-actin-like protain2
Actin, alpha skeletal muscle	Casein kinase cell subunit alpha
ADP/ATP translocase 4	Spartin
Actin, gamma-enteric smooth muscle (Fragment)	Actin, gamma-enteric smooth muscle (Fragment)
Actin, cytoplasmic 1 (Fragment)	Nucleoredoxin
Beta-actin-like protein 2	**Keratin 8**
Actin, gamma-enteric smooth muscle	Alpha globin 1
Histone H1.2	**Type ll Collagen, alpha-1**
Superoxide dismutase (Cu–Zn)	Hemoglobin subunit beta-2
Actin, aortic smooth muscle	Ubiquitin-405 ribosomal protein S27a
Alpha globin 1	Myelin protein P0
14–3-3 protein sigma	ADP/ATP translocase 4
ATP synthase subunit delta, mitochondrial	Histone H1.4
**Keratin 76**	Actin, cytoplasmic 1 (Fragment)
Hemoglobin subunit beta-2	Creatine kinase M-type
Histone H3.3C	Splicing factor 3A subunit 3
Actin, cytoplasmic 2	Cluster of Myelin protein P0
Tropomyosin alpha-1 chain	Myelin peripheral protein
Malate dehydrogenase, mitochondrial	Cluster of Creatine kinase M-type
Nuclear pore complex protein Nup85	Actin, alpha skeletal muscle
Histone H1.4	Actin, aortic smooth muscle

Proteomes were established for 6 samples each of naïve Biozzi mouse brain and spinal cord meninges. Data was filtered to include only those proteins present in at least 4/6 samples. The 25 most prominent proteins present in each category are listed. Notably, bolded proteins *Type II collagen, alpha-1* and *Type II keratins* are represented in brain (keratin 76) and spinal cord (keratin 8)

**Fig. 1:**
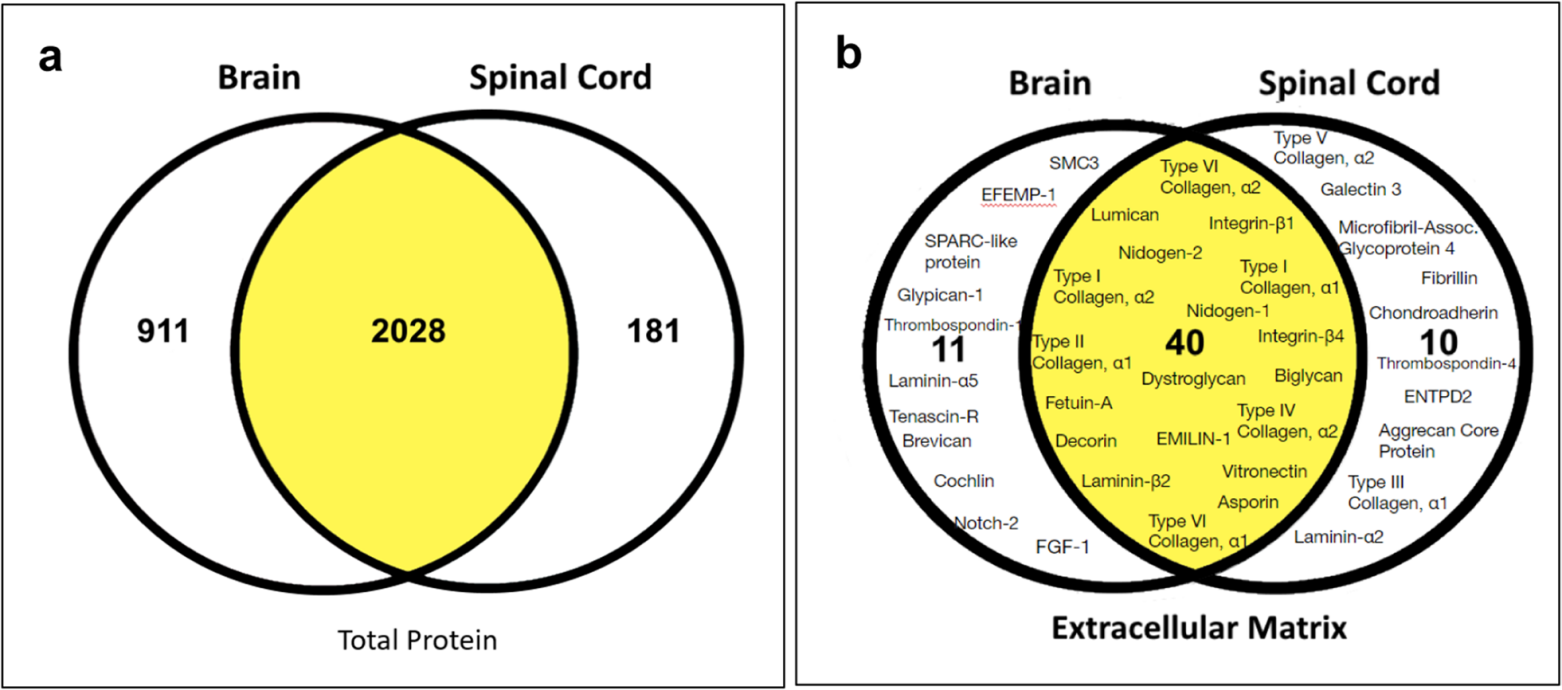
Shared and unique proteins in brain and spinal cord (SC) meninges. Proteomes were established from naïve Biozzi mice (Additional file 1: Fig. S3) and comparisons made between brain and spinal cord meninges. **a** *Total Proteins*. A total of 2027 proteins were shared between the two meningeal locales, 911 proteins unique to brain and 181 unique to spinal cord. **b** Comparison and distribution of specific *Extracellular Matrix* proteins. This category was expanded as it most likely contributes to the meningeal trabeculae. Note, not all 40 shared *Extracellular Matrix* proteins are listed

The vast majority of total proteins detected were shared between brain and spinal meningeal compartments (Fig. 1a). Overall, 2028 proteins were found in both domains, with 911 proteins being detected uniquely in brain, and 181 proteins exclusively noted in spinal tissue. Upon further subdividing total proteins by Cellular Compartment, the majority of proteins in each compartment were similarly found in both brain and spinal meninges (Additional file 1: Fig. S2). However, as in the case of total proteins, brain meninges generally contained considerably more “unique” proteins when compared across compartments. Differing from this pattern was the Extracellular Matrix category, which is most likely to reflect structural components of the SAS. Brain and spinal meninges each had nearly an equal number of “unique” Extracellular Matrix proteins (Fig. 1b), with two collagens, Type V and Type III, registering exclusively in the spine according to the proteomics criteria used. A similar restriction of Type III collagen to human spinal meninges was also previously noted [74]. Though not yet described in the meninges, Type V collagen is a form of fibrillar collagen found in association with tissues containing Type I collagen (Weis et al., 2010). In contrast to the selective expression of Types III and V collagen, multiple α-chains of Type I, Type II, and Type VI collagen were shared between brain and spinal meninges.

**Fig. 2:**
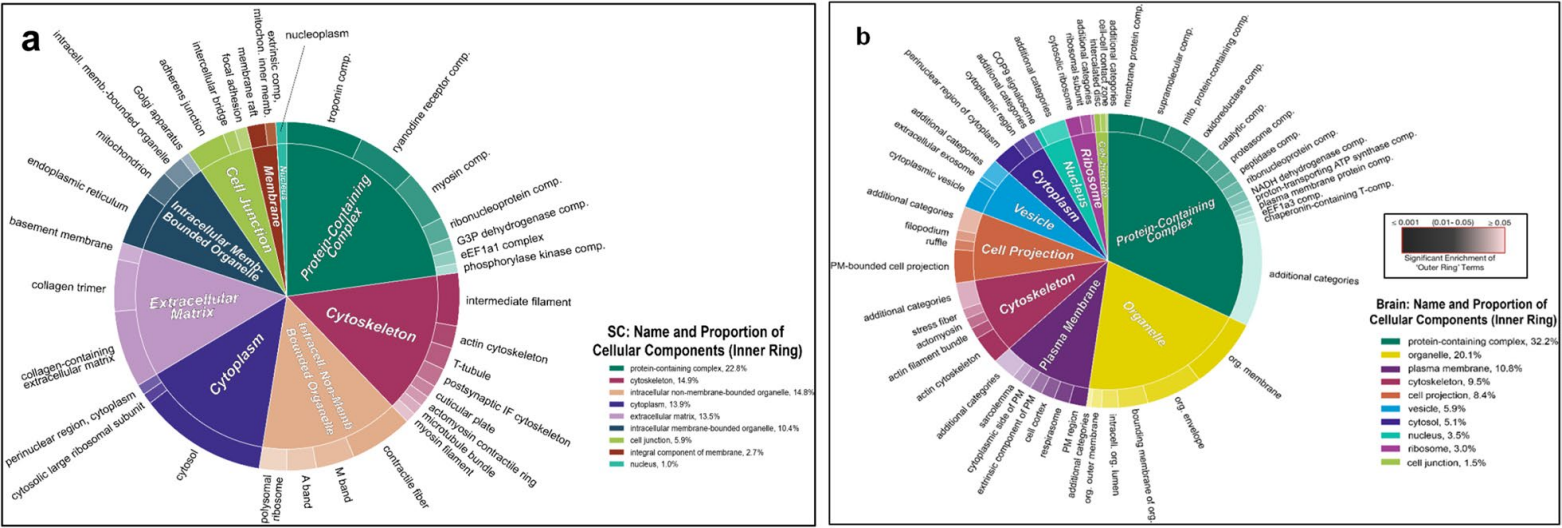
CirGo plots of highly enriched Gene Ontology (GO) terms in meninges. Proteomes were subject to qualitative enrichment analysis. **(a)** Spinal cord (“SC”) meninges. **(b)** Brain meninges. The inner circle slices represent broad (“parent”) GO Cellular Compartment clusters, while the outer ring represents specific GO (‘child’) terms that fall within each parent cluster. Color gradients emphasize the largest to smallest value distribution within the outer ring child terms

To next examine differences in the overall protein profile of brain and spinal meninges, respectively, two-dimensional pie charts were generated using the Python package CirGO in combination with the web based ReViGO server [59, 115]**.** While proteins of the spinal meninges showed less diversity of enriched inner-circle parent categories and outer-ring child terms, they were significantly enriched in those mapping to *Extracellular Matrix* and *Cell Junction* parent categories (Fig. 2a). Major derivative terms for the *Extracellular Matrix* category included “collagen trimer” and “collagen-containing extracellular matrix,” reflecting the expression of the varied collagen peptides. And the *Cell Junction* category encompassed “adherens junction” and “focal adhesion” terms, consistent with reports of E-cadherin-expressing cells in the arachnoid membrane [31, 32]. The brain meninges, by comparison, failed to record enrichment of the *Extracellular Matrix* category by this analysis (Fig. 2b). Though the brain meningeal proteome did contain extracellular matrix proteins (Fig. 1b), these were not of sufficient proportion to be considered enriched. However, the brain meninges evidenced enrichment of the parent terms *Cytoskeleton* and *Cellular Projections,* along with the derivative child terms “plasma membrane-bounded cell projections.” There are thus appreciable differences in brain vs spinal meninges with regard to selective enrichment of proteins serving structural functions.

**Fig. 3:**
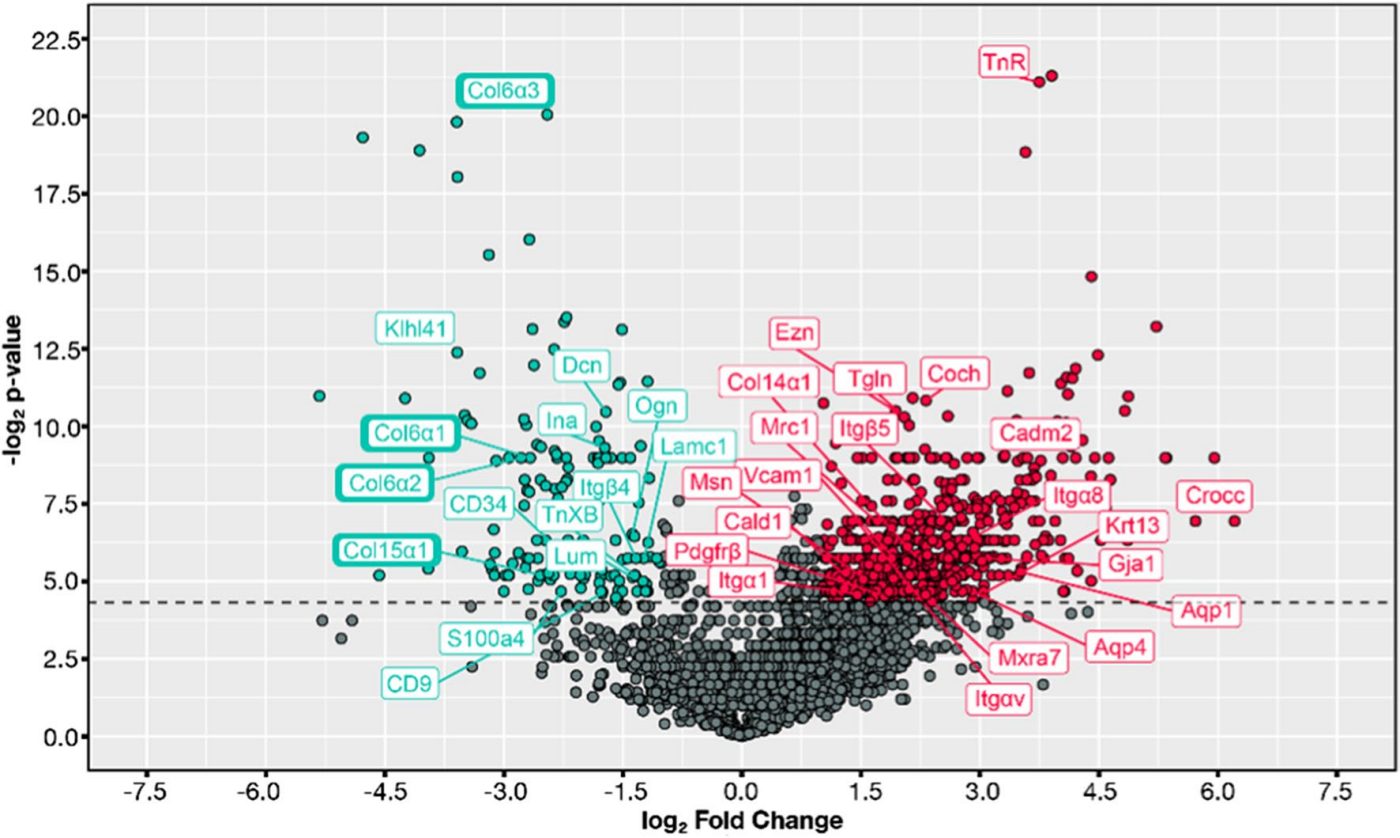
Volcano plot of differential expression in brain vs spinal cord meningeal proteomes. The quantitative proteomics data was used to determine individual proteins upregulated in brain vs spinal cord meninges. Upregulation refers to an increase in the magnitude of protein abundance in one compartment vs the other (log_2_(Fold Change) Brain/Spinal Cord). Colored points above the dotted line indicate statistically significant upregulation. Several, but not all, differentially expressed proteins are identified. Select ECM proteins upregulated in spinal cord meninges are specifically highlighted in bold boxes

**Fig. 4:**
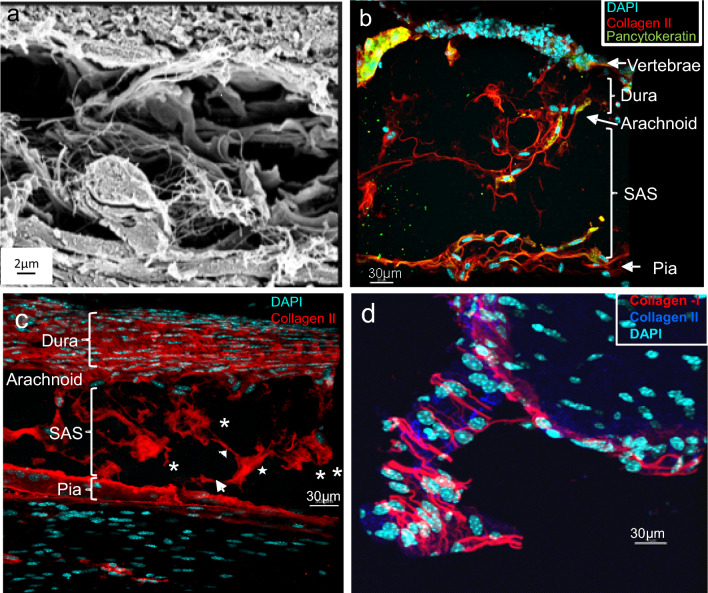
Immunofluorescence detection of trabeculae. (**a**) SEM of rat meninges, performed as described (Saboori, [111]), highlighting reticular network of diverse trabeculae. (**b**)Immunofluorescence of mouse spinal meninges, carried out on a tissue section cut from intact mouse spinal column and transferred to adhesive tape as described in Methods. Structural features of spinal meninges align with near superimposition to those in the SEM, highlighting staining of trabeculae by anti-collagen II and pan-cytokeratin antibodies. (**c**) Collagen II staining of mouse spinal meninges highlights *plate-like* (✻), *tree-like* ( ←), *veil-like* (✭)*,* and *rod-like* (◀) trabeculae seen in SEM (Saboori, [111]), as well as the dura*.* **(d)** Mouse optic nerve meninges indicating non-overlapping collagen I and collagen II staining

Following qualitative enrichment analysis of protein sub-category distribution, quantitative comparison of brain and spinal meningeal proteomes was performed. In order to determine if proteins were disparately expressed in either meningeal locale, fold change of protein abundance levels in brain and spinal cord were calculated (Fig. 3). The majority of proteins observed in either compartment showed no significant difference in expression. However, a greater number of brain meningeal proteins were significantly upregulated when compared to those of the spinal cord. The top 25 proteins upregulated in brain and spinal cord meninges, respectively, are listed in Table 2. The full listing of proteins differentially expressed between the two meningeal locales is shown in Additional file 3: Table S4. Notable structural proteins prominently upregulated in brain meninges include tenascin-R (Fig. 3**, **Table 2) and Keratin 13 (Fig. 3). Tenascin-R (TnR) is an extracellular matrix glycoprotein exclusive to the CNS [8], where it affects cell migration, adhesion and differentiation, and has been localized in human fetal brain meninges by the end of the third trimester [35]. Keratin 13 (Krt13), a type I keratin [104], is considered a marker of non-keratinized squamous epithelium, and has yet to be described in normal meninges. Though overall fewer in number, proteins significantly upregulated in spinal cord are largely glycoproteins and fibril-forming collagens, complementing the enriched representation of the “collagen-containing extracellular matrix” and “collagen trimer” sub-categories in the pie-chart analysis. In particular, several collagens were significantly upregulated in the spinal meninges (Fig. 4a), namely α1, α2, and α3 chains of Type VI collagen, and the α1 chain of Type XV collagen. Type VI collagen expression has been described in the meninges previously, where it is found in the basal lamina of meningeal vessels [40]. Likewise, Type XV collagen has been reported around blood vessels in the brain, as well as along the pial interface separating the brain parenchyma from the extra parenchymal tissues (meninges and blood vessels) [30].

**Table 2:** Top 25 significantly upregulated proteins in brain and spinal cord meninges

Brain	Spinal Cord
Solute carrier family 22 member 6	Myotilin
Sideroflexin-5	Nebulin
Brevican core protein	Type VI collagen, alpha 3
Solute carrier family 12 member 5	AMP deaminase 1
Folate receptor alpha	Myomesin 2
Autotaxin (ENPP2)	Myomesin 1
Band 4.1-like protein 1	Tripartite motif-containing protein 72
Sodium-coupled neutral amino acid transporter 3	Ryanodine receptor 1
OCIA domain-containing protein 2	SH3 domain-binding glutamic acid-rich protein
Phosphatidylinositol transfer protein beta isoform	Kelch-like protein 40
Tenascin-R	Cluster if Ryanodine receptor 1
Neuronal growth regulator 1	Fatty acid-binding protein, adipocyte
Synapsin-2	Phosphoglucomutase-1
Inward rectifier potassium channel 13	Neurofilament medium polypeptide
Neurotrimin	Kelch-like protein 41
Opioid-binding protein cell adhesion molecule-like	Carbonic anhydrase 3
Cluster of Synapsin-1	Myosin-binding protein C, fast-type
Cluster of Opioid-binding protein cell adhesion molecule-like	Alpha-actinin-2
Cluster of Sodium-driven chloride bicarbonate exchanger (NDCBE)	Cluster of Fatty acid synthase
Haloacid dehalogenase-like hydrolase domain-containing protein 3 (HDHD3)	Fatty acid synthase
Sodium-driven chloride bicarbonate exchanger (NDCBE)	Calsequestrin-1
Metallothionein-3	Myozenin-1
4-aminobutyrate aminotransferase, mitochondrial	Decorin
Cochlin	Myosin-binding protein C, slow-type

Proteins significantly upregulated in each compartment vs the other are listed in descending order of log2 p-value

### Histological localization of meningeal proteins

The localization of several structural proteins was next established by immunofluorescence and aligned with the proteomic data. Significantly, structural features revealed by fluorescence aligned spatially with those seen by SEM, allowing for molecular identification of external morphology (Fig. 4a, b).Type II collagen, one of the most abundant proteins detected proteomically in both brain and spinal meninges, localized most obviously to the pia in addition to cellular and extracellular elements of the SAS (Fig. 4b). Some staining also appeared along the arachnoid and what might be the innermost layer of the dura (Fig. 4c). Images depict what appear to be Type II collagen fibrils bundled in varying sizes and shapes, and forming a scaffold onto which cells of the SAS are wrapped, the two elements forging the trabecular meshwork connecting the arachnoid and pia (Fig. 4b, c). This is in agreement with prior descriptions of transmission electron micrographs characterizing the SAS as containing extracellular collagen closely associated with “arachnoid trabecular cells” [41, 117]. In particular, trabeculae have been reported as composed of collagen bundles and “leptomeningeal cells” [4], as well as denoted as “collagen-reinforced material” stretching between the arachnoid and pia membranes (Mortazavi et al. [75]). Scanning electron micrographs have also imparted “collagen fibrils constitute the internal structure” of trabeculae [99]. The web-like nature of Type II collagen was accentuated following isosurface rendering of immunostained trabeculae (Additional file 1: Fig. S3).

Type I collagen—also detected in both brain and spinal meninges by proteomics—failed to show consistent localization at either locale, despite evaluation with four different antibodies to the subunit protein Col1A1. The reason for this irregularity is unclear, and might reflect that Type I collagen is only present in low amounts in highly discrete meningeal domains, e.g., a distinct subpopulation of trabeculae or SAS structures along the brain and spinal cord, and thus not reliably detectable by less sensitive immunohistology. In fact, our proteomic assessment found Type I collagen to be repeatedly lower abundant than Type II collagen. Both Type I and Type II collagen did, however, show robust immunofluorescence localization in the meninges of the optic nerve (Fig. 4d), in what are suggestive of trabecular structures, septa or pillars previously described by transmission (Anderson, [7]) and scanning electron microscopy [54] at this locale and containing collagenous fibrils. Notably, the fluorescence patterns were not overlapping, suggesting different collagens might prevail at different meningeal sites and form distinct trabeculae or other retiform elements in the SAS. This is in agreement with previous findings of considerable “structural variability” within the meninges depending on location along the optic nerve [54], and variations of the spinal SAS “from one segmental level to the next within the same specimen” [76]. In a similar vein, the volume fraction attributable to arachnoid trabeculae, as determined by optical coherence tomography, has been described as “significantly region-dependent in the cerebrum” [12].

**Fig. 5:**
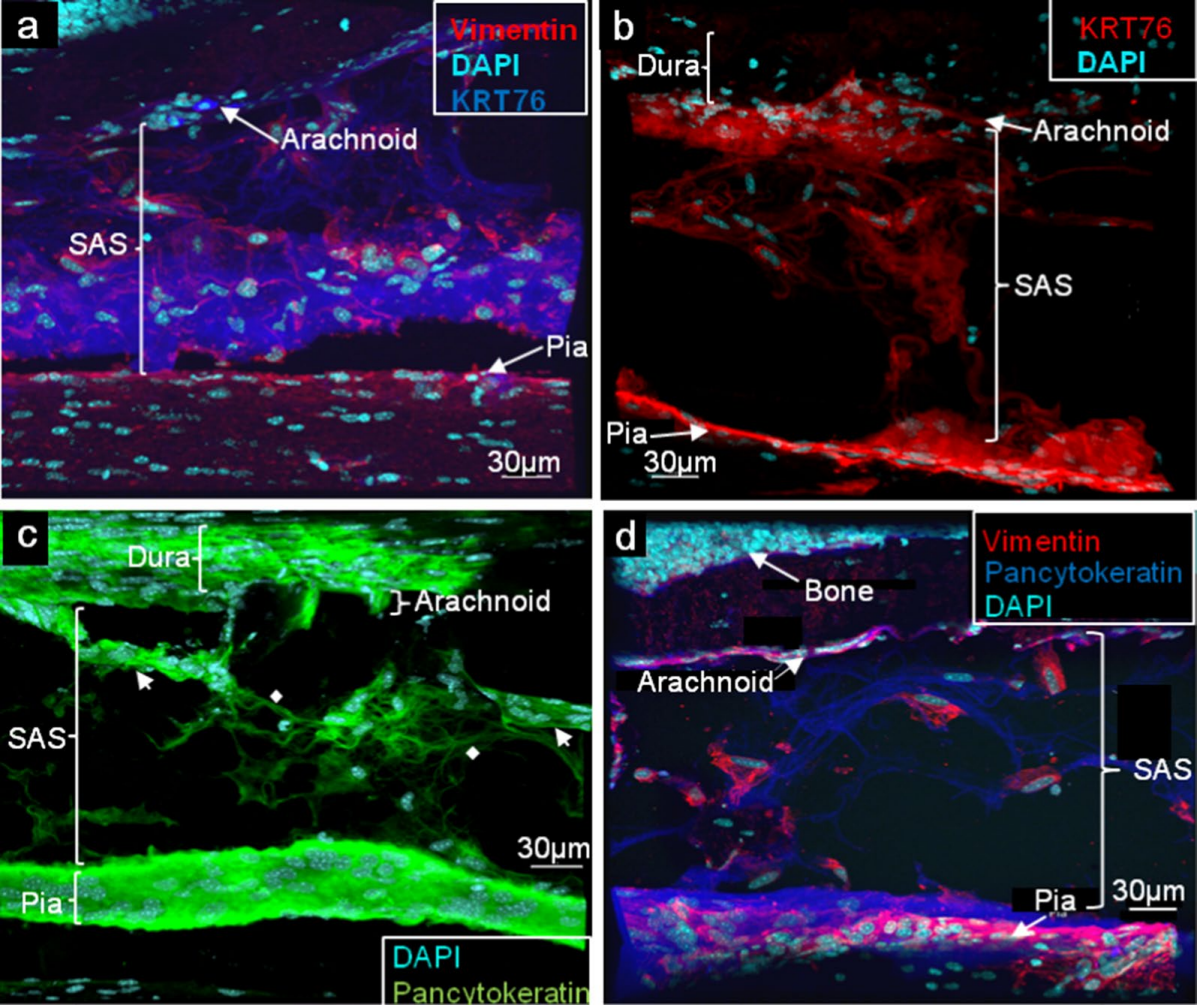
Vimentin and cytokeratin identify trabecular and other meningeal cells. Immunofluorescence of mouse spinal meninges, performed as in Fig. 4. **(a)** Vimentin staining highlights cells within the SAS associated with trabeculae, as well as a thin layer of pial cells most closely apposed to the SAS. Keratin 76 (KRT76) staining is also seen in cells of the pia and arachnoid, as well as diffusely throughout the trabecular mesh, but does not overlap with vimentin. **(b)** Keratin 76 is associated with sheets of trabecular cells in addition to pial and arachnoid membranes**. (c)** Pancytokeratin staining of cellular sheets ( ←), characteristic of epithelial cells; cytokeratin-filled, cell processes (◆) may contribute to trabeculae of the *filform* type [4]. **(d)** Pancytokeratin staining of cells in the pia and arachnoid, and filamentous processes in the SAS; vimentin highlights cell bodies associated with trabeculae

The meninges also showed clear and reproducible immunostaining of the SAS and trabeculae with antibodies against vimentin and cytokeratins. Vimentin, the Type III IF protein expressed by mesenchymal cells (Damjanov et al. 1982), localized to trabecular cells, as well as cells of the pia and arachnoid (Fig. 5a, d; Additional file 1: Fig. S4). This agrees with previous reports of immunohistochemical detection of vimentin in embryonic [13], normal [4] and pathologic human meningeal tissue [44, 79]. Vimentin staining – particularly of trabecular cells—was clearly distinguished from the neighboring Type II collagen staining, however immunoreactivity of both proteins was evident in cells of the arachnoid and, to a lesser extent, in the pia (Additional file 1: Fig. S4). Keratin 76, prominently detected during proteomic assessment, was also widely distributed within the pia, arachnoid, and cells of the trabecular meshwork, though in non-overlapping manner with vimentin (Fig. 5a, b). Certain cells, however, appeared to display staining of both filamentous proteins, in accord with description of leptomeningeal tissue as manifesting both fibroblastic and epithelial qualities [31, 128]. Staining with pancytokeratin antibody, which detects multiple cytokeratins, likewise highlighted cells in the pia and arachnoid (Figs. 4b, 5c, d), as well as cells traversing the SAS that appear in singular form in association with thin, Type II collagen + filamentous structures (Fig. 4b) or in sheets (Fig. 5c). These distinct filamentous and sheet-like forms possibly represent filiform trabeculae/chordae and membranous septa, respectively, described in ultrastructural studies [4, 83]. Some cells also appeared positive for both pancytokeratin and Type II collagen staining (Fig. 4b). Of note, pancytokeratin staining of the pia appeared to highlight a wider swath of membrane than did keratin 76 staining (contrast Figs. 5a, c, d), possibly suggesting that keratins in addition to keratin 76 contribute to pial composition.

Brain meninges were also analyzed to show they, too, remain intact through our tissue sectioning protocol (Additional file 1: Fig. S5). Staining of Type II and Type III collagen was evident at a region near the cerebral-cerebellar border (Additional file 1: Fig. S5a). The patterns were clearly distinct, but multiple cells appeared to express both proteins. Though Type III collagen was considered one of the proteins of the Extracellular Matrix category to be exclusive to the spinal meninges (Fig. 1b), this was based solely on the thresholding criterion of needing to be detected in 4/6 samples. Type III collagen was only detected in 3/6 samples of brain meninges and, thus, not considered reproducibly present at this local. Staining of tenascin-R, which was one of the Extracellular Matrix proteins that registered unique to the brain meninges, seemingly localizes along the pial interface at the parenchymal surface, as well as within the subarachnoid space (Additional file 1: Fig. S5b).

Imaging Mass Cytometry (IMC) was used as another platform to resolve Type I collagen localization, in the off chance that immunofluorescence posed unique and unrecognized staining challenges for brain or spinal meninges. As seen in spinal section (Additional file 1: Fig. S6), collagen I staining was largely confined to bone, though immunoreactivity along the pia was also seen. No patent staining of trabeculae or other structures within the SAS was observed.

### Ultrastructural localization of meningeal proteins

**Fig. 6:**
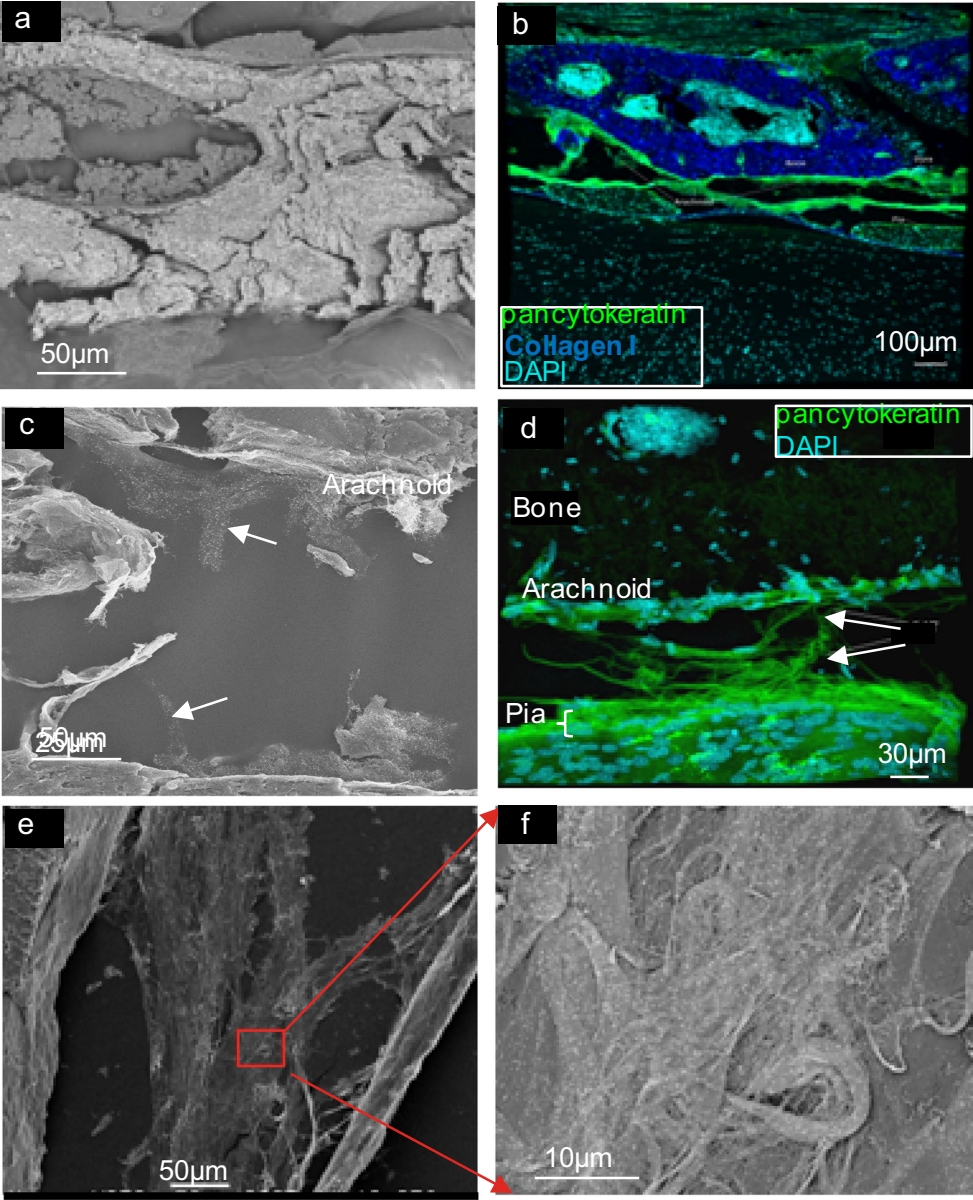
Immuno-SEM of meninges. Sections of normal mouse spinal meninges adhered to adhesive tape, and processed for immuno-SEM, or immunofluorescence/confocal microscopy as described in Methods. **(a)** Immuno-SEM of collagen I staining (backscatter mode), showing 1 nm gold labeling (white dots) localized to the bony vertebrae. **(b)** Immunofluorescence of collagen I exclusively staining the bony vertebrae overlying the meninges; the dura and arachnoid membranes show intense pancytokeratin staining. **(c)** Immuno-SEM of pancytokeratin staining (backscatter mode), showing 1 nm gold staining (white dots) of what appear to be trabecular sheets (arrows); gold labeling can be seen extending toward the arachnoid membrane. (**d)** Immunofluorescence of pancytokeratin staining, highlighting similar sheets of trabeculae as seen in SEM, as well as arachnoid and pial membranes. **(e)** Low magnification immuno-SEM (secondary electron mode) of collagen II staining, highlighting sheet-like forms of trabeculae. **(f)** High magnification immuno-SEM of collagen II staining (backscatter mode), showing enlarged region (red box) in (e) containing 1 nm gold labeling (white dots)

To further examine protein localization at higher resolution, tissue samples were examined using immuno-scanning electron microscopy (immuno-SEM). The pia, arachnoid, dura, SAS with its varied forms of trabeculae and other traversing structural elements, and attached vertebral bone were evident in spinal samples (Additional file 1: Fig. S7). Findings further replicated observations made by immunofluorescence **(**Fig. 6, Additional file 1: Fig. S8). Gold-labeling of Type II collagen was observed in association with varied trabecular forms, including sheet-like (Fig. 6e, f) and filiform **(**Additional file 1: Fig. S8) structures. Type I collagen staining was not observed in the meningeal tissue, but only in the surrounding vertebrae (Fig. 6a, b), where it was consistently detected across samples. Pancytokeratin immunoreactivity was also confirmed at the ultrastructural level (Fig. 6c), reflecting the staining seen in immunofluorescence (Fig. 6b, d).

### Confirmation of meningeal protein expression

Lastly, Western blotting was used to confirm expression of some prominent structural proteins that did not show reproducible staining patterns by immunofluorescence or immuno-SEM (Additional file 1: Fig. S9), as well as to reinforce earlier quantitative assessment. Type I collagen α1 peptide was detected at similar levels in both brain and spinal meningeal samples, which aligned with the proteomic results. In agreement with the volcano plot analysis, Type VI collagen was present in higher amount in the spinal meninges compared to meninges of the brain. Keratin 8—among the 15 most abundant proteins expressed in spinal meninges (Fig. 1)—likewise was in greater abundance in spinal meninges by western blot. However, Type III collagen, which was only detected in 3/6 samples and, thus, didn’t satisfy the 4/6 criterion for being considered present in brain meninges (Fig. 1b), showed similar levels in both meningeal compartments. This was most likely the result of Type III collagen being just below the limit of proteomic identification reproducibility in the brain meningeal samples.

## Discussion

Proteomic interrogation provided a first-hand accounting of the molecular nature of prominent structural components of the meninges, revealing several proteins that had not yet been identified in the meninges. It additionally illuminated potential differences that exist in this tissue between the brain and spinal cord. (Fig. 1, Table 1). Specifically, meninges of the brain displayed a greater number and diversity of proteins. Enrichment analysis allowed for a ‘bird’s eye view’ of both proteomes, showing differential and diverse function in cellular components and protein expression (Figs. 2 – 4 and Table 2). Expression and localization of prominent structural proteins were further confirmed by immunostaining at the fluorescence microscopic and ultrastructural levels (Figs. 5, 6), assigning protein identities to meningeal features previously described mainly morphologically by electron microscopy. Western blotting further validated proteomic evaluation, in some cases reinforcing examples of differential protein expression (Additional file 1: Fig. S9).

Notwithstanding protein validation by several means, the prospect of contamination of meningeal samples was a valid concern. It need be underscored that perfusion of Evan’s blue dye before dissection was critical in making the large meningeal vessels stand out, clearly differentiating the meninges from the underlying parenchymal tissue and overlying bone. Specifically, the entire meninges, even outside the large vessels, retained a distinct blue hue, while the parenchyma and skull, in stark comparison, were left off-white. This overt difference was due to the larger macrovasculature of the meninges binding considerably more dye than did the decidedly smaller microvasculature of the extra-meningeal tissue, and significantly facilitated distinguishing the meninges from their surrounds. Moreover, clarifying the meningeal extracts by spinning through 0.22 μm centrifuge filters excluded small bone chips, further limited bone contamination. Detection of tight junction protein, claudin-11 (Additional file 1: Fig. S3), which has been localized to arachnoidal cells (Uchida et al., 2019), but not other tight junction proteins characteristic of parenchymal vessels, reinforces that contamination was kept to a minimum. Despite the preventative steps taken, it is acknowledged that elimination of contaminating extra-meningeal tissue could not be completely assured.

The abundance of collagen proteins was expected, as arachnoid trabeculae have been described as “a system of branching and anastomosing collagen bundles” (Anderson, [7]), and a “spongy connective tissue made of collagen fibers and fibroblasts” [29]. Ultrastructural analysis has further revealed “the SAS containing collagen fibrils in longitudinal and cross section [26]. Less anticipated in our findings was the preeminence of Type II collagen, which has restricted distribution and predominates in the hyaline cartilage of articular surfaces [11, 65], where it provides tensile strength by resisting to swelling pressure [60]. Transcripts (Col2A1) from the Type II collagen gene have been detected at low level in human fetal brain [53], with concentration in the meninges [64, 100]. But, until now, there has been no documentation of which we are aware of this gene being expressed in adult meninges or of Type II collagen protein localized to this tissue at the fetal or adult stage. In the present studies, Type II collagen protein was expressed in meninges of both brain and spinal cord, histologically localizing to both sites and within those of the optic nerve as well. It also manifested immunostaining patterns consistent with descriptions of diverse trabecular structures and other elongated elements within the SAS that connect the arachnoid and pial membranes (Anderson, [4, 7, 22, 23, 54, 77, 83]. The elaborate lattice appearance of Type II collagen in the SAS offers the prospect of providing a trap and/or support for immune cells migrating into the meninges from the calvarial and vertebral bone marrow [27, 43], peripheral circulation [105], or CSF [92]. Type II collagen might further cooperate with other collagens to organize leukocytes with the SAS. For example, Type III collagen, also found in the meninges, can cross-link the surface of Type II collagen and modify its fibril network [125]. As Type III collagen forms the reticulin fibers that organize lymph nodes [46, 58], it, together with Type II collagen as a support, might establish an analogous reticular network in the meninges to retain and/or segregate leukocyte populations during neuroinflammation [71]. The appearance in the SAS of specialized immune cell aggregates called tertiary lymphoid organs (TLOs,also referred to, among other names, as tertiary lymphoid structures and B cell follicles during progressive phases of the demyelinating, neuroinflammatory disease Multiple Sclerosis and its animal model, experimental autoimmune encephalomyelitis (EAE [107, 129] might designate such a capacity. In this case, leukocytes trapped in a web of arachnoid trabeculae would be an example of compartmentalized inflammation [50]. Such a prospect is supported by descriptions of reticular networks in association with or enveloping immune cell aggregates within the meninges of mice with EAE [68], Pikor et al. [87]).Type II collagen has additionally been recognized as a major target for peripheral autoimmune responses [38, 103] and exhibits a known common splice variant that binds TGFß [131]. Thus, besides acting in a structural capacity, it might also serve as substrate for and/or determinant of meningeal inflammatory activity in CNS autoimmunity [70]. Type VI collagen, found in both brain and spinal meninges, consists of monomers that aggregate linearly to form beaded filaments or laterally through their globular domains [9], and might further lend to creating a 3D network in these tissues capable of filtering cellular and soluble elements [56].

Particularly surprising, however, was that Type I collagen was not observed in the brain or spinal meninges by immunofluorescence or immuno-SEM. As both these immunostaining techniques did detect considerable distribution of this protein in the surrounding bone, antibody reactivity was not problematic. It is further unlikely that the observed meningeal staining by anti-Type II collagen actually reflected covert Type I collagen reactivity, as none of four different anti-Type I collagen antibodies evaluated reproducibly stained the meninges. These findings are seemingly at odds with assertions that cranial arachnoid trabeculae are “predominantly made of Type I collagen” [12], and may call into question prior recognition of this protein by non-biochemical methods. In this regard, its presence in the adult meninges has largely been implied from structural features revealed by electron microscopy (Mortazavi et al. [75], Saboori, [111]), second harmonic generation imaging [24], and polarized light microscopy [74], with confirmation of its expression restricted to developing meningeal fibroblasts [32, 51]. According to transmission electron micrographs, “the periodicity seen with alternating light and dark periods'' confirmed the presence of this particular fibrillar collagen (Mortazavi et al., [75]). Perhaps the periodical D-band pattern, which is generally recognized as a unique ultrastructural characteristic shared by all fibril-forming collagens [80], lent to this assumption. Distinctive negative-staining band patterns displayed by Type II collagen could potentially resolve this matter [81]. And in a lone report listing histological immunopositivity of Type I collagen in fetal and adult meninges, no supporting microscopic images accompanied this classification [74].

Equally notable to the absence of immunodetectable Type I collagen from sections of adult mouse brain and spinal meninges, was its overt histological expression, along with Type II collagen, in meninges of the optic nerve. Such regional variability in collagen expression in the meningeal landscape could reflect a diversity in functional performance of this tissue along the CNS axis. Moreover, that the Col1A1-GFP transgenic marker has been reported to label a diverse population of regionally segregated meningeal fibroblasts in E14 mouse embryos [32] might further indicate Type I collagen plays a more prominent role during embryonic development than in adulthood. A few non-mutually exclusive possibilities may be considered for the seemingly inconsistent detection of Col1A1by proteomics and Western blot, but not microscopy. The prominence of Type I collagen in the walls of large blood vessels, particularly arteries [93, 127], and the tenuous attachment of these structures to the leptomeninges, could be contributing factors. While these vessels would be retained in samples directly solubilized for biochemical analysis, they could have been dislodged when tissue sections were repeatedly washed during immunostaining—leading to Col1A1 appearance in the former but not the latter. Alternatively, as Col-1 contributes to 90% of the total organic component of bone matrix [21], the Col1A1peptide detected in meningeal proteomes of both brain and spinal cord could have been due to minor contamination from various bone cell populations or extracellular collagen that accompanied scraping of the dura from skull and vertebrae [102]. Despite its absence from trabeculae, Type I collagen may be associated with the pia, as reflected by IMC analysis. The detection by IMC, alone, of Type I collagen along the pial membrane might reflect the unique sensitivity of this technique and/or application of the specific metal conjugated antibody.

Aside from various collagens, IF-forming keratin and vimentin proteins were prominently detected and distributed throughout the pia, arachnoid and SAS. Type II keratins are basic or neutral, high molecular weight (50—70 kD) fibrous proteins expressed in epithelial cells [37, 45], wherein they perform a scaffolding function while affording resistance to stress and damage [72]. Keratin 76, in particular, is required for barrier-forming tight junctions in skin squamous epithelial cells [34] and, in what might portend a role in CNS autoimmunity, has been shown to possess immunomodulatory properties [108]. Keratin filaments and desmosomes have also been described in “arachnoidal sheaths” of some higher vertebrates [2], and keratin 8 immunoreactivity detected in cells of normal human spinal leptomeninx [48] and various meningiomas [69]. By comparison to other cytoskeletal and structural proteins, IFs possess unique viscoelasticity properties [20], enabling them to control viscous dissipation of energy (Bonifasi-Lista et al., 2005). This feature may contribute to meninges lowering the potential for brain or spinal injury following traumatic incidents [91]. The presence of both keratin and vimentin proteins is supportive of the dual characterization of meningothelial cells as being at once “specialized epithelial cells” [94] and “fibroblast-like cells” [128]. While keratins are conventionally recognized as the IF-forming subunits of epithelial cells (Karantza, [47]), and expression of these proteins taken as evidence of a cell’s epithelial origin (Werner et al., [122]), there have been reports of keratin gene and protein expression, along with that of vimentin, in cells of mesenchymal derivation, including fibroblasts [49, 116]. In the present study, patterns of keratin 76 and vimentin distribution were non-overlapping but, in some cases, both proteins appeared to be expressed by the same cell. Thus, some meningothelial cells might be in a transitional state between fibroblast and epithelial type. That arachnoid cells can change between epithelial and mesenchymal states during meningeal reconstruction following injury [18] further conveys such a transitional nature of the meningothelial population. While most of the collagen staining was extracellular in the images shown here, some cells appeared immunoreactive for both pancytokeratin and Type II collagen, implying a proportion of meningeal collagen might originate from cells with epithelial qualities. Though contrary to the prevailing opinion that fibroblasts are the predominant collagen-producing cell type [39, 52], this agrees with prior findings that collagens Type I and II are secreted by epithelial cells in corneal tissue [42].

The observed differences between the brain vs spinal cord meningeal proteomes echo previous assertions of segmental variation in microsurgical anatomy within the SAS and region-dependent diversity in the density and morphology of trabecular structures [12, 74, 76]. From the histological perspective, most striking was the exclusive distribution of Type I collagen within the meninges of the optic nerve, where it took the form of bundles suggestive of the trabeculae and pillars previously described at this CNS level [54]. What significance this heterogeneity holds is only a matter of speculation at this time. But, given the increasing role attributed to the meninges as a cradle of immune activity (Russi and Brown, [22, 23, 66, 95, 96], Di Marco Barros et al. [33, 14], such disparities in structural and ECM proteins might provide a basis for differences in the neuroinflammatory response between the brain and spinal cord [106, 130]. Reflecting this possibility is the finding that resident, meningeal fibroblastic stromal cells undergo remodeling during EAE, in a process directed by infiltrating Th17 cells and accompanied by production of ECM, recruitment of inflammatory cells, and establishment of TLOs Pikor et al. [87, 88]. Though it is unknown if the identity of these particular stromal cells is the same as the keratin^+^ and/or Type II collagen^+^ cells associated with trabecular structures in the present study, what is clear is the reticulum of meningothelial cells and derivative ECM has the potential to establish immune cell niches in the CNS and influence the course of neuroinflammation [16, 87, 88, 90]. In turn, organization and regulation of specific immune cell populations by the meninges could stem, in part, from the ability of epithelial and fibroblastic cells to secrete chemokines and/or provide leukocyte binding sites [113], Shaykhiev and Bais, [109, 119]. It is further significant that meningeal stromal cells, in particular, can release proinflammatory cytokines and inflammatory mediators [87, 123], that foster and modulate focal leukocyte habitats in the SAS.

Several elaborate efforts employing single-cell analysis to interrogate its immune cell repertoire have laid bare that the meninges—far from a historical lone protective role—is an immunological niche of considerable complexity [16, 57, 97, 101, 102, 118]. Future work to compare changes in the composition and arrangement of the meningeal landscape at the brain and spinal levels during neuroinflammatory episodes could shed light on how and why inflammatory foci develop where they do, and bring the meninges into the drug orbit of therapeutics targeting MS and other neurodegenerative diseases (Russi and Brown, [61, 66, 96].

### Supplementary Information

**Additional file 1:  Fig. S1**. Removal of meninges from mouse brain and spinal cord. Following initial perfusion with PBS to remove blood, Evans Blue dye was perfused to stain the meningeal vessels. (Top row) Brain, with meninges containing stained blood vessels. (a) Before removal of brain meninges. (b) After removal of meninges from the left side of the brain; the right side of the brain shows meninges still adherent. (Bottom row) Spinal cord. (c) Before removal of meninges. (d) After removal of meninges from the caudal end of the spinal cord (left side of image); spinal meninges are shown still adherent to the rostral end.** Fig. S2**. Compartmental analysis of shared and unique proteins in brain and spinal cord (SC) meninges. Total Proteins from proteomes in Figure 2 were further subdivided into the respective subcellular compartments and numbers of shared and unique proteins within each compartment of brain and spinal cord meninges indicated. **Fig. S3**. Meningeal trabeculae form a 3D meshwork. Iso-surface rendering (Imaris software) of collagen II-stained trabeculae, highlighting a dense meshwork that permeates the subarachnoid space. This could act to filter leukocytes in the CSF, and aid in nucleating formation of mELTs. As lymphoid chemokines can bind collagen peptides, trabeculae may help set-up concentration gradients necessary to drive immune cell recruitment.** Fig. S4**. Vimentin and collagen II show divergent networks in the SAS. Collagen II staining within normal mouse spinal meninges casts a diffuse net through the SAS, and is distinct from the focal vimentin staining highlighting trabecular cells. Select cells of the arachnoid and pial layers exhibit staining of both structural proteins.** Fig. S5**. Immunofluorescence of brain meninges. Sections were cut through whole mouse skull, at a region near the junction of the cerebrum and cerebellum, and adhered to adhesive tape. Brain meninges remain intact as in sections through spinal column (Figs. 4 and 5). (a) Staining of collagen II and collagen III within an apparent dural fold. (b) Staining of ECM protein, tenascin-R, and cytoskeletal protein, vimentin.** Fig. S6**. IMC of meninges. A section of normal mouse spinal meninges adhered to adhesive tape and processed by IMC as described in Methods. Metal-conjugated antibodies (collagen I and MHC II) and a cationic nucleic acid intercalator containing natural abundance iridium 191Ir and 193Ir) were used. Collagen staining is most prominent in vertebral bone but is also seen along the pia. MHC II identifies some antigen presenting cells in the vertebral bone marrow, while the DNA intercalator highlights a high density of nuclei in the bone.** Fig.S7**. SEM of subarachnoid trabeculae. Sections of normal mouse spinal meninges adhered to adhesive tape and processed for SEM as described in Methods, highlighting different shaped trabeculae with attached structures. (a) Filiform (←), rod-like (✻), and tree-like (◂) trabeculae. (b) Sheet-like structures (←) between the bone (B), dura (DM) and pia (PM), the latter having been torn away from the underlying parenchyma. (c) Veil-like trabeculae (✻) and what may be trabeculae-associated cell bodies (←).** Fig. S8**. Immuno-SEM of collagen II. Sections of normal mouse spinal meninges adhered to adhesive tape and processed for immuno-SEM as described in Methods.** Fig. S9**. Western blotting of meningeal proteins. Western blotting was carried on a sampling of meningeal proteins to compare with proteomic and immunohistological results. Gel images highlighting the respective protein bands are depicted on the left, and corresponding quantification of protein bands is shown on the right. Boxes are denoted for ease of grouping like samples together. For each protein being assessed, three samples each of brain and spinal cord meninges, respectively, were run on the same gel and blotted onto the same membrane. (a) Type III collagen; (b) Type VI collagen; (c) Keratin 8; (d) Type I collagen.** Table S1**. Primary antibodies used for immunofluorescence, immune-SEM, and Western.** Table S2**. Secondary antibodies used for immunofluorescence.

**Additional file 2:  Table. S3**. Total proteins detected in brain and spinal meninges. Proteomic analysis was performed on 6 samples each of naïve Biozzi mouse brain and spinal.

**Additional file 3:  Table S4**. All proteins significantly upregulated in brain and spinal cord meninges.

## Data Availability

Data will be made available upon reasonable request.
