# Supplementary material for: Proteomic interrogation of the meninges reveals the molecular identities of structural components and regional distinctions along the CNS axis
Source: Fluids Barriers CNS. 2023 Oct 19;20:74. doi: 10.1186/s12987-023-00473-w (PMC10588166; doi:10.1186/s12987-023-00473-w)
Supplement: Supplementary file 1 — Additional file 1: Fig. S1. Removal of meninges from mouse brain and spinal cord. Following initial perfusion with PBS to remove blood, Evans Blue dye was perfused to stain the meningeal vessels. (Top row) Brain, with meninges containing stained blood vessels. (a) Before removal of brain meninges. (b) After removal of meninges from the left side of the brain; the right side of the brain shows meninges still adherent. (Bottom row) Spinal cord. (c) Before removal of meninges. (d) After removal of meninges from the caudal end of the spinal cord (left side of image); spinal meninges are shown still adherent to the rostral end. Fig. S2. Compartmental analysis of shared and unique proteins in brain and spinal cord (SC) meninges. Total Proteins from proteomes in Figure 2 were further subdivided into the respective subcellular compartments and numbers of shared and unique proteins within each compartment of brain and spinal cord meninges indicated. Fig. S3. Meningeal trabeculae form a 3D meshwork. Iso-surface rendering (Imaris software) of collagen II-stained trabeculae, highlighting a dense meshwork that permeates the subarachnoid space. This could act to filter leukocytes in the CSF, and aid in nucleating formation of mELTs. As lymphoid chemokines can bind collagen peptides, trabeculae may help set-up concentration gradients necessary to drive immune cell recruitment. Fig. S4. Vimentin and collagen II show divergent networks in the SAS. Collagen II staining within normal mouse spinal meninges casts a diffuse net through the SAS, and is distinct from the focal vimentin staining highlighting trabecular cells. Select cells of the arachnoid and pial layers exhibit staining of both structural proteins. Fig. S5. Immunofluorescence of brain meninges. Sections were cut through whole mouse skull, at a region near the junction of the cerebrum and cerebellum, and adhered to adhesive tape. Brain meninges remain intact as in sections through spinal column (Figs. 4 and 5). (a) Staining [file 12987_2023_473_MOESM1_ESM.docx]

**Additional file 1: Fig. S1. Removal of meninges from mouse brain and spinal cord.** Following initial perfusion with PBS to remove blood, Evans Blue dye was perfused to stain the meningeal vessels. (Top row) Brain, with meninges containing stained blood vessels. **(a)** *Before* removal of brain meninges. **(b)** *After* removal of meninges from the left side of the brain; the right side of the brain shows meninges still adherent. (Bottom row) Spinal cord. **(c)** *Before* removal of meninges. **(d)** *After* removal of meninges from the caudal end of the spinal cord (left side of image); spinal meninges are shown still adherent to the rostral end.

**Additional file 1: Fig. S2**. **Compartmental analysis of shared and unique proteins in brain and spinal cord (SC) meninges.** *Total Proteins* from proteomes in Figure 2 were further subdivided into the respective subcellular compartments and numbers of shared and unique proteins within each compartment of brain and spinal cord meninges indicated.

**Additional file 1: Fig. S3. Meningeal trabeculae form a 3D meshwork.** Iso-surface rendering (Imaris software) of collagen II-stained trabeculae, highlighting a dense meshwork that permeates the subarachnoid space. This could act to filter leukocytes in the CSF, and aid in nucleating formation of mELTs. As lymphoid chemokines can bind collagen peptides, trabeculae may help set-up concentration gradients necessary to drive immune cell recruitment.

**Additional file 1: Fig. S4. Vimentin and collagen II show divergent networks in the SAS.** Collagen II staining within normal mouse spinal meninges casts a diffuse net through the SAS, and is distinct from the focal vimentin staining highlighting trabecular cells. Select cells of the arachnoid and pial layers exhibit staining of both structural proteins.

**Additional file 1: Fig. S5. Immunofluorescence of brain meninges.** Sections were cut through whole mouse skull, at a region near the junction of the cerebrum and cerebellum, and adhered to adhesive tape. Brain meninges remain intact as in sections through spinal column (Figs. 4 and 5). **(a)** Staining of collagen II and collagen III within an apparent dural fold. **(b)** Staining of ECM protein, tenascin-R, and cytoskeletal protein, vimentin.

**Additional file 1: Fig. S6. IMC of meninges.** A section of normal mouse spinal meninges adhered to adhesive tape and processed by IMC as described in Methods. Metal-conjugated antibodies (collagen I and MHC II) and a cationic nucleic acid intercalator containing natural abundance iridium 191Ir and 193Ir**)** were used. Collagen staining is most prominent in vertebral bone but is also seen along the pia. MHC II identifies some antigen presenting cells in the vertebral bone marrow, while the DNA intercalator highlights a high density of nuclei in the bone.

**Additional file 1: Fig.S7. SEM of subarachnoid trabeculae.** Sections of normal mouse spinal meninges adhered to adhesive tape and processed for SEM as described in Methods, highlighting different shaped trabeculae with attached structures. **(a)** Filiform (←), rod-like (✻), and tree-like (◀) trabeculae. **(b)** Sheet-like structures (←) between the bone (B), dura (DM) and pia (PM), the latter having been torn away from the underlying parenchyma. **(c)** Veil-like trabeculae (✻) and what may be trabeculae-associated cell bodies (←).

**Additional file 1: Fig. S8. Immuno-SEM of collagen II.** Sections of normal mouse spinal meninges adhered to adhesive tape and processed for immuno-SEM as described in Methods.


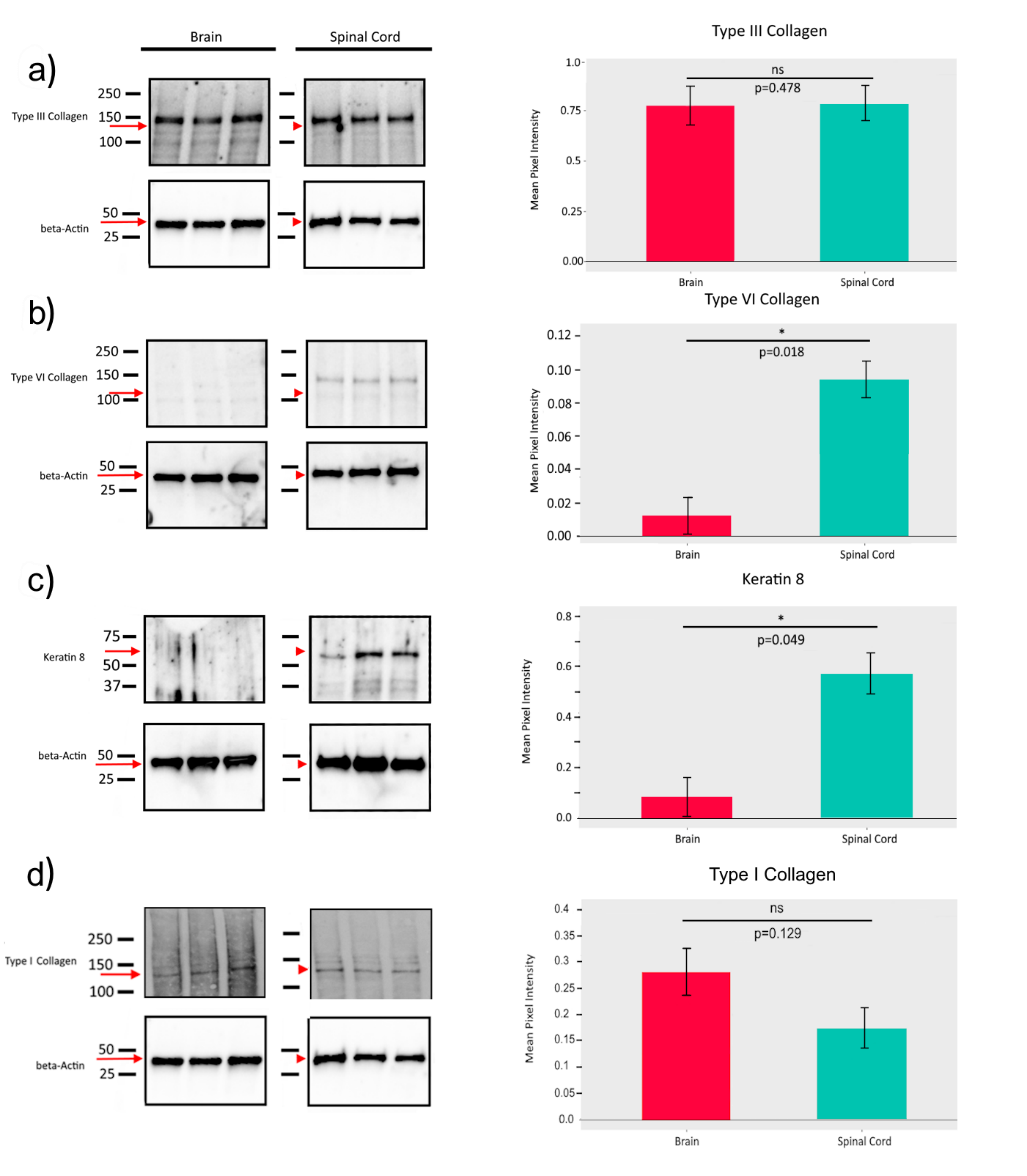


**Additional file 1: Fig. S9. Western blotting of meningeal proteins.** Western blotting was carried on a sampling of meningeal proteins to compare with proteomic and immunohistological results. Gel images highlighting the respective protein bands are depicted on the left, and corresponding quantification of protein bands is shown on the right. Boxes are denoted for ease of grouping like samples together. For each protein being assessed, three samples each of brain and spinal cord meninges, respectively, were run on the same gel and blotted onto the same membrane. **(a)** Type III collagen; **(b)** Type VI collagen; **(c)** Keratin 8; **(d)** Type I collagen.

**Additional file 1: Table S1. Primary antibodies used for immunofluorescence, immune-SEM, and Western blotting.**

|  | Dilution | Species | sources | Cat. # |  |
| --- | --- | --- | --- | --- | --- |
| Antibody for immunostaining  Type I Collagen, polyclonal Antibody | 1:150 | Goat | Chemicon®, via Millipore Sigma | AB758 |  |
| Type I Collagen, polyclonal antibody | 1:150 | Rabbit | Abcam | ab21286 |  |
| Type I Collagen, alpha 1 propeptide | 1:150 | Rabbit | Rockland Antibodies and Assays | 600-401-D19 |  |
| Type I Collagen, monoclonal Antibody | 1:150 | Mouse | Invitrogen | MA1-26771 |  |
| Type II Collagen, monoclonal antibody | 1:150 | Mouse | Invitrogen | MA5-13028 |  |
| Type III Collagen monoclonal antibody | 1:150 | Mouse | Invitrogen | MA1-22147 |  |
| Cytokeratin, pan monoclonal antibody | 1:150 | Mouse | Sigma-Aldrich | C2931 |  |
| Keratin K3/K76 , monoclonal antibody | 1:150 | Mouse | EMD Millipore Sigma | CBL218 |  |
| Tenascin R, monoclonal antibody | 1:150 | Mouse | R&D Systems | MAB1624 |  |
| Vimentin, polyclonal antibody | 1:150 | Chicken | Invitrogen | PA1-10003 |  |
| Antibody for Western Blotting |  |  |  |  | |
| Type I Collagen, Monoclonal Antibody | 1:000 | Goat | Chemicon®, via Millipore Sigma | AB758 | |
| Type III Collagen, Monoclonal Antibody | 1:000 | Rabbit | Invitrogen | MA5-42628 | |
| Type VI Collagen, Polyclonal Antibody | 1:000 | Rabbit | Proteintech via ThermoFisher | 17023-1-AP | |
| Cytokeratin 8, Polyclonal Antibody | 1:000 | Rabbit | Invitrogen | PA5-29607 | |
| Beta-Actin, polyclonal | 1:000 | Rabbit | Invitrogen | PA5-16889 | |

**Additional file 1: Table S2. Secondary antibodies used for immunofluorescence.**

|  | Dilution | Species | sources | Cat. # |
| --- | --- | --- | --- | --- |
| Anti-Rabbit IgG (H+L), Alexa Fluor 488 | 1:150 | Goat | Life Technologies | A11034 |
| Anti-Mouse IgG (H+L), Alexa Fluor™ 488 | 1:150 | Donkey | Invitrogen | A21202 |
| Anti-Chicken IgY (H+L), Alexa Fluor 555 | 1:150 | Goat | Life Technologies | A21437 |
| Anti-Rabbit IgG (H+L), Alexa Fluor™ 555 | 1:150 | Goat | Invitrogen | A21428 |
| Anti-Mouse IgG (H+L), Alexa Fluor™ 555 | 1:150 | Donkey | Invitrogen | A31570 |
| Anti-Goat IgG (H+L), Alexa Fluor™ 555 | 1:150 | Donkey | Invitrogen | A21432 |
| Anti-Mouse IgG (H+L), Alexa Fluor™ 633 | 1:150 | Goat | Invitrogen | A21053 |
| Anti-Mouse IgG (H+L), Alexa Fluor™ 647 | 1:150 | Donkey | Invitrogen | A31571 |
| Anti-Goat IgG (H+L), Alexa Fluor™ 647 | 1:150 | Donkey | Invitrogen | A21447 |
